# Xanthogranulomatous endometritis

**DOI:** 10.52054/FVVO.15.4.106

**Published:** 2023-12-13

**Authors:** A Morales Vicente, Y García Sánchez, N Santonja López, J Gilabert Estellés

**Affiliations:** Department of Obstetrics and Gynaecology, Hospital General Universitario de Valencia. Avenida Tres Cruces, 2, 46014, Valencia, Spain; Department of Anatomy and Histology, Hospital General Universitario de Valencia. Avenida Tres Cruces, 2, 46014, Valencia, Spain

**Keywords:** Hysteroscopy, endometritis, pyometra, endometrial cancer

## Abstract

Xanthogranulomatous endometritis (XGE) is an uncommon inflammatory benign condition that can mimic endometrial cancer. The majority of the reported cases of XGE have been observed in postmenopausal women, often presenting clinically as haematometra or benign senile pyometra. We report a case of XGE in a 73-year-old woman who presented with pyometra. Diagnostic hysteroscopy is an important tool when accompanied by endometrial samples for histology in suspected cases. Knowledge of this uncommon disease is crucial for accurate diagnosis. XGE is a benign condition, however, there have been reported cases of chronic active XGE and bacterial infection in which hysterectomy was required due to complications.

## Introduction

Xanthogranulomatous endometritis (XGE) is a rare inflammatory chronic benign condition associated with endometrial hyperplasia and endometrial carcinoma. Clinically, it can mimic endometrial malignancy (Barua et al., 1978; Makkar et al., 2013). There are few reported cases in the worldwide literature with the majority occurring in postmenopausal women who may also have endometrial hyperplasia, endometrial carcinoma and/or cervical stenosis (Barua et al., 1978; Na et al., 2020).

The pathogenesis of XGE still remains under debate (Russack and Lammers,1990; Zhang et al., 2012). The various causative factors implicated are chronic inflammation associated with pyometra (Zhang et al., 2012; Malik et al., 2019;), diabetes (Na et al., 2020) or tumour necrosis induced by radiotherapy in the endometrium or cervix (Du et al., 2019). Diagnostic hysteroscopy allows obtaining endometrial samples to rule out malignant pathology (Song et al., 2019). We report the case of XGE in a 73-year-old woman who presented with pyometra.

## Case Report

A 73-year-old postmenopausal female who complained of continuous yellow fluid discharge per vaginum attended the department. The patient had a history of one normal delivery and menopause at the age of 54. She had no history of endometriosis, pelvic inflammatory disease, or use of any intrauterine device. Her family history was unremarkable.

The patient had previously undergone three normal hysteroscopies in 2008, 2015, and 2019 due to suspicion of an endometrial polyp. Haematological and biochemical investigations were normal. Cytology revealed an atrophic smear.

Transvaginal ultrasonography showed a collection in endometrial cavity suggestive of pyometra/ hematometra. Endometrial thickness was increased (11 mm) and it was irregular. Attempts to perform endometrial sampling were unsuccessful as the cannula could not pass through the cervix.

**Figure 1:**
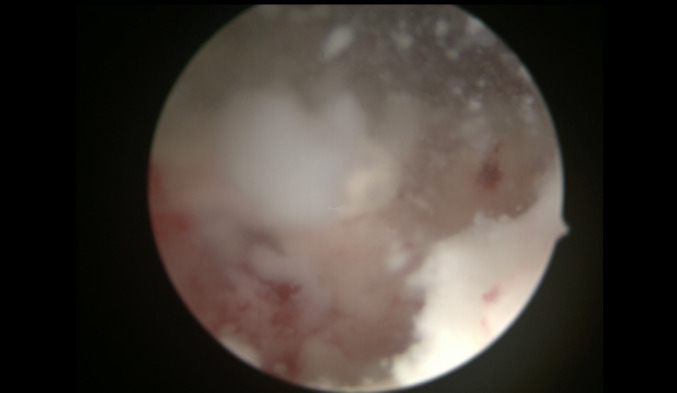
Endometrial cavity filled with mucus and thickened endometrium.

Given the cervical stenosis and thickened endometrium with pyometra in an elderly female, diagnostic hysteroscopy was performed to evaluate the endometrial cavity and obtain endometrial biopsy. Vaginoscopy revealed synechiae and fibrosis in the upper vaginal third prior to the cervix. The atrophic cervix showed severe stenosis. Uterine endometrial cavity was dilated and filled with thick cottony mucus presenting fundal fibrotic central synechia. The endometrium was atrophic and irregular. Drainage of mucus was performed, and endometrial biopsy specimens were obtained for histology (Figure 1).

**Figure 2:**
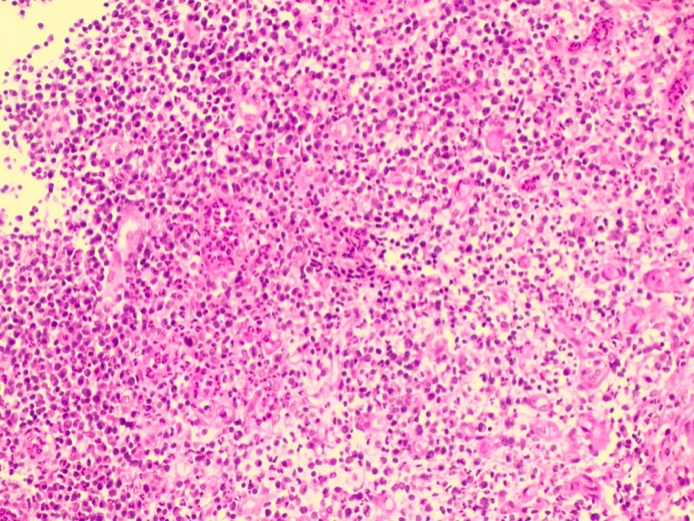
H&E 10x. Endometrium with abundant vascularization and dense inflammatory infiltrates, resembling granulation tissue. Neoplasic endometrial tissue is not seen. Abundant foamy histiocytes with neutrophils, plasma cells and lymphocytes. Endometrial glands are not seen.

**Figure 3:**
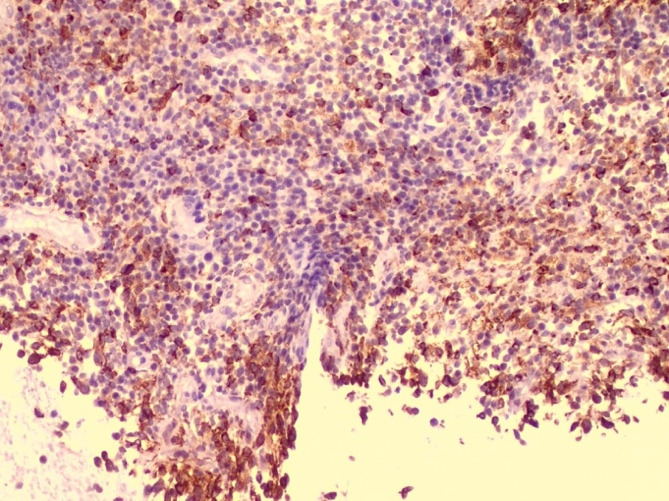
CD68 10x. Presence of abundant foamy histiocytes with CD68 positive marker within the endometrium.

On microscopic examination, Haematoxylin and Eosin-stained sections showed abundant foamy histiocytes (CD68 positives) mixed with siderophages, neutrophils, lymphocytes, and plasma cells. There was no evidence of endometrial hyperplasia or endometrial carcinoma. With the above findings, a histological diagnosis of XGE was made (Barua et al., 1978) (Figures 2 and 3).

The final diagnosis was consistent with pyometra with XGE, secondary to cervical stenosis. The patient is currently asymptomatic and under regular follow-up. Written consent was taken from the patient for article submission.

## Discussion

XGE also known as histiocytic endometritis or pseudoxanthomatous endometritis is an extremely rare chronic inflammatory benign condition, which may be associated with endometrial hyperplasia and endometrial carcinoma (Russack and Lammers, 1990; Doǧan-Ekici et al., 2007).

It is extremely rare with just over 30 cases reported in the wider literature (Zhang et al.,2012; Du et al., 2019). It typically occurs in postmenopausal women who may have endometrial hyperplasia, endometrial carcinoma and/or cervical stenosis (Barua et al., 1978; Na et al., 2020). It can also occur after radiotherapy-induced endometrial or cervical tumour necrosis (Na et al., 2020).

Xanthogranulomatous is a type of chronic inflammation characterised by foamy lipid laden histiocytes mixed with other inflammatory cells (Na et al., 2020). The most common sites of xanthogranulomatous inflammation are the kidneys and the gallbladder. XGE is extremely rare (Du et al., 2019).

The pathogenesis of XGE still remains debatable (Russack and Lammers,1990; Zhang et al., 2012). The various causative factors implicated are chronic inflammation associated with pyometra due to postmenopausal cervical stenosis or cervical carcinoma (Zhang et al., 2012, Malik et al., 2019; Du et al., 2019). Diabetes has been reported as a risk factor for xanthogranulomatous inflammation in the gallbladder, kidneys, and testis due to a leukocyte dysfunction common in diabetic patients (Du et al., 2019).

Bacteria may or may not be isolated, but when related to bacterial infection, the involved organisms include Escherichia coli, Proteus vulgaris, Peptostreptococcus magnus, or Enterococcus spp (Na et al., 2020).

XGE is a benign condition, however there are reported cases of chronic active XGE and bacterial infection in which hysterectomy was required due to complications with signs of systemic inflammation and septic shock (Na et al., 2020).

Differential diagnoses of XGE include malakoplakia, pseudo decidual change of endometrial stroma or Langerhans cell histiocytosis (LCH). In Malakoplakia, also a rare entity of the female reproductive tract, exists the pathognomonic Michaelis-Gutmann bodies which can exclude this diagnosis. Pseudodecidualized stroma should be CD10 positive. In XGE, histiocytes are CD68 positive while in LCH, they are CD1a and langerin (CD207) positive (Na et al., 2020; Du et al., 2019).

XGE may mimic endometrial malignancy (Doǧan-Ekici et al., 2007). The most common radiologic feature is a heterogeneous cystic uterine mass, however radiological and clinical examination alone are not enough to establish the diagnosis. Histological examination is essential for diagnosis and exclude the mimickers (Malik et al., 2019).

Hysteroscopy is an important diagnostic tool in cases of endometritis especially when accompanied by cervical stenosis, as it allows endometrial sampling and the exclusion of malignant pathology.

## Conclusion

XGE is a very rare pathology with benign characteristics that mimics endometrial carcinoma both clinically and radiologically. Direct hysteroscopic examination and histological examination is essential to establish the diagnosis and rule out malignancy. Most of cases of XGE might resolve spontaneously or with antibiotic treatment, however, in some cases it may progress to peritonitis or sepsis with a poor prognosis or coexist with malignant pathology (Na et al., 2020).

## References

[1] Barua R, Kirkland JA, Petrucco OM (1978). Xanthogranulomatous endometritis: case report.. Pathology.

[2] Doǧan-Ekici AI, Usubütün A, T Küçükali (2007). Xanthogranulomatous endometritis: A challenging imitator of endometrial carcinoma.. Infect Dis Obstet Gynecol.

[3] Du X-Z, Lu M, Safneck J (2019). Xanthogranulomatous endometritis mimicking endometrial carcinoma: A case report and review of literature.. Radiol Case Report.

[4] Makkar M, Gill M, Singh D (2013). Xanthogranulomatous endometritis: an unusual pathological entity mimicking endometrial carcinoma.. Ann Med Health Sci Res.

[5] Malik V, Chatterjee D, Goel B (2019). Xanthogranulomatous Endometritis: A Benign Uncommon Masquerader of Malignancy.. J Midlife Health.

[6] Na JM, Kim MH, Ko GH (2020). Xanthogranulomatous endometritis: a report of two Korean cases with cytologic findings.. J Pathol Transl Med.

[7] Russack V, Lammers RJ (1990). Xanthogranulomatous endometritis. Report of six cases and a proposed mechanism of development. Arch Pathol Lab Med.

[8] Song D, Li TC, Zhang Y (2019). Correlation between hysteroscopy findings and chronic endometritis.. Fertil Steril.

[9] Zhang XS, Dong HY, Zhang LL (2012). Xanthogranulomatous inflammation of the female genital tract: Report of three cases.. J Cancer.

